# Supplementary material for: Voriconazole Cyclodextrin Based Polymeric Nanobeads for Enhanced Solubility and Activity: In Vitro/In Vivo and Molecular Simulation Approach
Source: Pharmaceutics. 2023 Jan 24;15(2):389. doi: 10.3390/pharmaceutics15020389 (PMC9968121; doi:10.3390/pharmaceutics15020389)
Supplement: Supplementary file 1 [file pharmaceutics-15-00389-s001.zip › pharmaceutics-2047421-supplementary.pdf]

Supplementary Materials

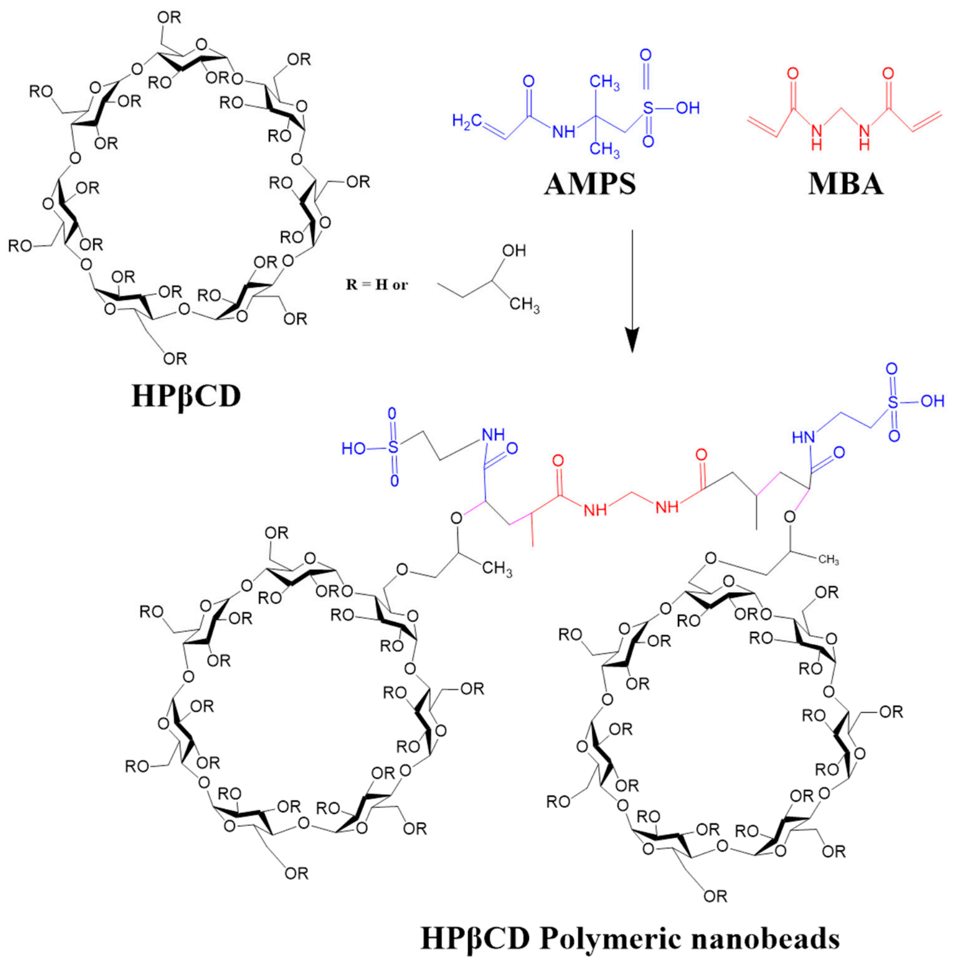

Supplementary Figure S1: Proposed Chemical Reaction of Hydroxy propyl β Cyclodextrin polymeric nanobeads

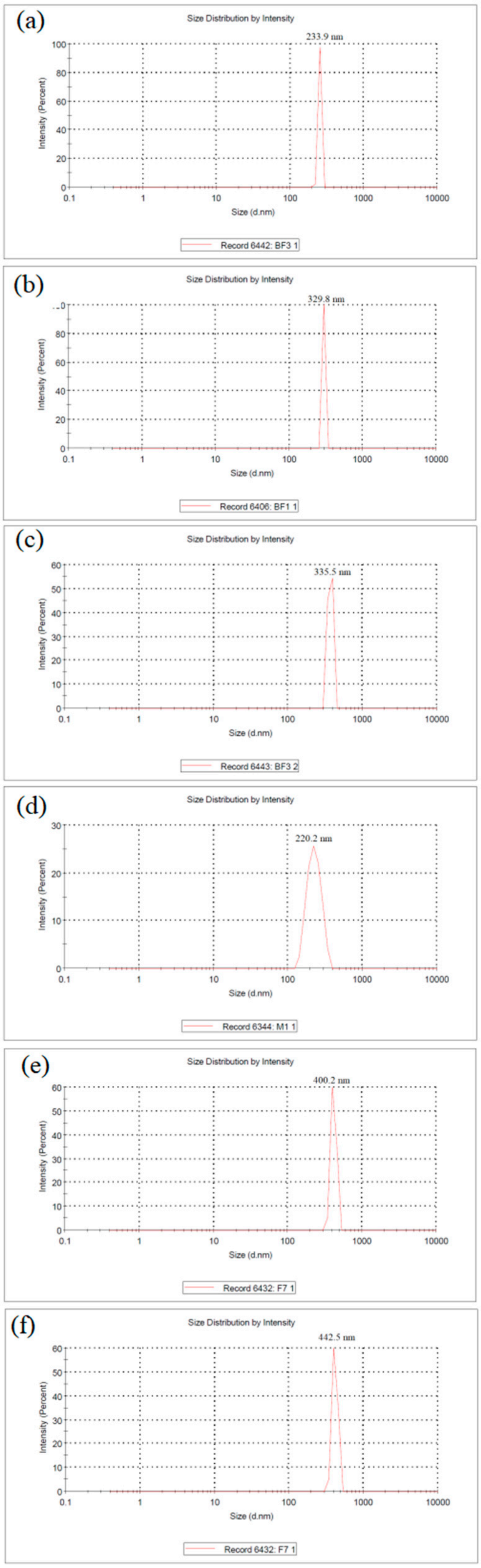

Supplementary Figure S2: Particle size of CDN1(a), CDN2 (b), CDN4 (c), CDN5 (d), CDN5 (e), CDN8 (f)

Supplementary Table S1: Biochemical Parameters (Mean ± SD, n = 5).

**Parameters**	**Group A**	**Group B**	**Group C**
Total Cholesterol (mg/dl)	48.00 ±0.03	47.80 ±0.07	47.88 ±1.09
ALT (IU/L)	63.03 ±0.11	63.26 ±0.02	63.24 ±0.08
AST (IU/L)	112.3 ±0.01	111.4 ±0.21	111.7 ±0.31
Serum creatine (mg/dl)	1.20 ±0.07	1.24 ±0.06	1.26 ±0.04
Triglycerides (mg/dl)	91.21 ±0.01	90.52 ±0.41	91.24 ±1.12
Serum urea (mg/dl)	4.91 ±0.08	4.78 ±0.09	4.87 ±0.04
Serum uric acid (mg/dl)	5.32 ±0.05	5.28 ±0.21	5.29 ±0.91
